# Traumatic stress, depression, and non-bereavement grief following non-fatal traffic accidents: Symptom patterns and correlates

**DOI:** 10.1371/journal.pone.0264497

**Published:** 2022-02-28

**Authors:** Paul A. Boelen, Maarten C. Eisma, Jos de Keijser, Lonneke I. M. Lenferink

**Affiliations:** 1 Department of Clinical Psychology, Faculty of Social Sciences, Utrecht University, Utrecht, The Netherlands; 2 ARQ National Psychotrauma Centre, Diemen, The Netherlands; 3 Department of Clinical Psychology and Experimental Psychopathology, Faculty of Behavioral and Social Sciences, University of Groningen, Groningen, The Netherlands; 4 Psychology, Health & Technology, Faculty of Behavioural, Management and Social Sciences, University of Twente, Enschede, The Netherlands; Public Library of Science, UNITED KINGDOM

## Abstract

Non-fatal traffic accidents may give rise to mental health problems, including posttraumatic stress (PTS) and depression. Clinical evidence suggests that victims may also experience grief reactions associated with the sudden changes and losses caused by such accidents. The aim of this study was to examine whether there are unique patterns of symptoms of PTS, depression, and grief among victims of non-fatal traffic accidents. We also investigated associations of emerging symptom patterns with sociodemographic variables and characteristics of the accident, and with transdiagnostic variables, including self-efficacy, difficulties in emotion regulation, and trauma rumination. Participants (N = 328, M_age_ = 32.6, SD_age_ = 17.5 years, 66% female) completed self-report measures tapping the study variables. Using latent class analysis (including symptoms of PTS, depression, and grief), three classes were identified: a no symptoms class (Class 1; 59.1%), a moderate PTS and grief class (Class 2; 23.1%), and a severe symptoms class (Class 3; 17.7%). Summed symptom scores and functional impairment were lowest in Class 1, higher in Class 2, and highest in Class 3. Psychological variables were similarly ordered with the healthiest scores in Class 1, poorer scores in Class 2, and the worst scores in Class 3. Different sociodemographic and accident related variables differentiated between classes, including age, education, and time since the accident. In a regression including all significant univariate predictors, trauma rumination differentiated Class 2 from Class 1, all three psychological variables differentiated Class 3 from Class 1, and difficulties with emotion regulation and trauma rumination differentiated Class 3 from Class 2. This study demonstrates that most people respond resiliently to non-fatal traffic accident. Yet, approximately one in three victims experiences moderate to severe mental health symptoms. Increasing PTS coincided with similarly increasing grief, indicating that grief may be considered in interventions for victims of traffic accidents. Trauma rumination strongly predicted class membership and appears a critical treatment target to alleviate distress.

## Introduction

Non-fatal traffic accidents may give rise to different types of mental health problems. Posttraumatic stress disorder (PTSD), depression, and anxiety disorders are among the most commonly studied mental health consequences of such incidents. Research shows that a significant proportion of victims are afflicted with these complaints [1, 2]. Both for theoretical and clinical reasons it is imperative to understand the wider range of mental health symptoms people may experience following such events. Clinical evidence suggests that people confronted with traffic accidents may experience other psychological reactions alongside anxiety and hypervigilance, implicated in traumatic stress, and low mood and lack of interest, implicated in both traumatic stress and depression. The current study focused on an additional category of reactions following such accidents, namely grief reactions.

There is increasing recognition that non-bereavement losses and other life-events may give rise to grief reactions (e.g., [3, 4]). Indeed, some traumatic events, including traffic accidents, can elicit significant loss experiences—including loss of health, future plans, and resources—that are impossible or difficult to undo. Just as the rupture in the bond with a loved one due to death can yield separation distress and grief, sudden changes in circumstances brought about by a traumatic event (including a traffic accident) may elicit grief reactions. For example, people may yearn for what is now gone, be preoccupied with memories of what was lost, and experience difficulties accepting what happened and implications thereof. Studying grief reactions associated with traumatic events has both theoretical and clinical relevance. Theoretically, understanding the phenomenological variety of emotional distress following such events can inform theorizing about mechanisms underlying the persistence of this distress [5]. From a clinical perspective, understanding the range of emotional reactions to traffic accidents and their underlying mechanisms can inform the development of treatment interventions.

The current study sought to expand existing knowledge on the psychological impact of non-fatal traffic accidents by examining individual variation in the experience of symptoms of posttraumatic stress (PTS), depression, and grief among victims of such accidents. To capture this variation, we used latent class analysis (LCA); LCA is a person-centred approach that allows for the identification of different homogenous subgroups (or latent classes) based on their scores on designated variables (i.e. PTS, depression, and grief reactions) [6]. We conducted LCA (as a person-centred approach) instead of categorizing people based on averaged scores on indices of PTS, depression, and grief (a variable-centred approach) because we were interested in the detection of possible subgroups characterized by different scores on these indices which is not possible using variable-centred approaches (e.g., [7]). We also preferred LCA over categorization based on scores above particular cut-offs (e.g., meeting versus not meeting criteria) because we included non-bereavement grief in our examination (for which no dichotomized classification exists). Moreover, LCA enables the identification of groups with subclinical psychopathology, which is not possible when categorizing people based on sample-based averaged scores or dichotomous cut-offs. We felt it was relevant to examine if subgroups with subclinical psychopathology could be identified, given that such subclinical psychopathology is associated with considerable distress and disability [8] and can be treated effectively [9].

Thus, the first aim of this study was to identify subgroups of people confronted with non-fatal traffic accident, based on their endorsement of items assessing symptoms of PTS, depression, and grief. At least two possible outcomes were anticipated. First, it was possible that parallel profiles would emerge, differentiated by increasing likelihoods of endorsing symptoms of PTS, depression, and grief. That would indicate that all symptoms cohere together such that, e.g., higher odds of experiencing PTS symptoms coincide with higher odds of experiencing depression and grief symptoms. Alternatively, it was possible that non-parallel profiles would emerge, differing in the endorsement of some, but not other symptoms. For instance, non-parallel profiles might reflect the presence of one subgroup with high PTS and grief and low depression and another subgroup with a reserved ordering of symptom severity. Such an outcome was considered possible considering different studies on emotional reactions to bereavement loss (e.g., [10, 11]) but also non-bereavement loss (e.g., job-loss; [12]) which have shown that these symptoms do not tend to cluster together but, instead, are differentially endorsed across subgroups.

A second aim was to clarify whether emerging subgroups differed in terms of functional impairment and different indices of mental health. There is evidence that different patterns of mental health symptoms after traumatic events are differentially related to functional impairment [13, 14]. Accordingly, we anticipated that participants included in classes with higher odds of experiencing symptoms would score higher both on measures of PTS, depression, and grief symptoms and measures assessing functional impairment.

We also sought to elucidate factors related to symptom profiles, focusing on both *static* and potentially *modifiable* variables. Specifically, our third aim was to explore to what extent emerging subgroups were associated with sociodemographic variables (i.e., age, level of education, gender) and characteristics of the accident (i.e., time elapsed since the accident, type of transportation during the accident, perceived threat to life, injury severity). Some of these characteristics, particularly perceived threat to life and injury severity, have been shown to be longitudinally associated with symptoms of PTSD and other negative health outcomes following traffic accidents [1, 2, 15]. It is conceivable that such factors are also associated with reactions of depression and grief. Elucidating factors related to higher odds of specific symptom patterns could help to identify survivors of traffic accident who are most at risk to develop mental health problems.

The fourth aim was to examine the role of three psychological, potentially modifiable variables in predicting the diversity in symptom presentation following non-fatal accidents, namely generalized self-efficacy, difficulties in emotion regulation, and trauma rumination. Self-efficacy is defined as a person’s perceived ability to achieve desired outcomes, even in the face of unexpected events [16]. Evidence shows that self-efficacy promotes adjustment to trauma and longitudinally predicts lower PTSD symptoms, possibly by fostering engagement in constructive coping efforts [17, 18]. It has also been found to be related to depression following stressful life events [19] and grief after bereavement [20]. Emotion regulation refers to efforts to affect the likelihood, intensity or duration of an emotion [21]; difficulties in emotion regulation include difficulties in identifying, understanding, and accepting negative emotions, access to regulation strategies, and abilities to pursue goal-directed behaviour and inhibit behavioural impulse when experiencing such emotions [22]. Research has established the linkage between such difficulties and PTSD, across different traumatized samples [23]. In addition, there is preliminary evidence that difficulties in emotion regulation are positively associated with grief [20]. Trauma rumination concerns abstract repetitive negative thinking about the traumatic event and its sequelae; it has been found to be longitudinally associated with symptoms of PTSD and depression following traffic accidents [1, 2, 24, 25]. Rumination exerts its negative impact by maintaining negative appraisals and interfering with the emotional processing of thoughts, feelings, and memories associated with the event [26]. We focused on these three psychological variables because they represent both potentially protective and maladaptive regulatory processes, are all transdiagnostic variables (i.e., related with different types of symptoms following adversity), and are potentially malleable to therapeutic intervention.

## Method

### Participants and procedure

The current study was part of the Dutch “TrafVic” project, developed to study the consequences of (deadly and non-deadly) traffic accidents for (bereaved and non-bereaved) victims of such accidents [27, 28]. The present study focused on psychological functioning of people confronted with non-deadly accidents. Recruitment occurred via announcements on internet websites, social media channels, letters sent to people who had been in contact with Victim Support (a non-profit organisation providing emotional and legal care for trauma victims) and peer support organisations, and university websites for students who could earn course credits for participation. Announcements explained the aims of the project and solicited people who had been involved in a traffic accident to participate by completing questionnaires online. People interested in participation could login to a secured online environment (programmed in Qualtrics), where more information about the study was given, informed consent could be provided, and questionnaire could be completed. To reduce the burden for participants, questionnaires were divided in two parts and participants were given the opportunity to discontinue completion of the questionnaires, after the first part. In total, 408 started completing the questionnaire. After removing people who terminated participation before completion of the measures of PTS, depression, and grief, excluding participants who had lost a loved one and who were referred to another study in the program, and people who filled in the questionnaire twice, data from 328 participants were used for the present study. All participants completed Part 1 of the questionnaires (including sociodemographic and accident-related variables and measures of PTS, depression, and grief); 296 also completed Part 2 (including measures of self-efficacy, emotion regulation, and trauma rumination). Consequently, analyses with these variables were based on n = 296. The ethics committee for psychological research from Groningen University approved the study (numbered: PSY-1819-S-0113). All participants provided written informed consent. A flowchart of participants, including details on recruitment source, is provided in **S1 Fig**.

### Measures

#### Sociodemographic characteristics

Participants were asked about their gender (dichotomized as 0 = male, 1 = female), age (in years), and education (multiple categories, collapsed into 0 = lower than college/university, 1 = college/university).

#### Characteristics of the accident

Participants reported the date of the accident and were asked what transportation type they used during the accident (categorized as described in Table 2) and whether they were the driver of the transportation vehicle (0 = no, 1 = yes). Drawing from prior research (e.g.,[15]), perceived threat to life was measured with a single item (“To what extent did you fear for your own life during the traffic accident?”) rated on a 7-point scale ranging from 1 = not at all, to 7 = a lot. Also drawing from prior work [29] the question “Were you physically injured in the accident?” was posed to obtain an index of injury severity, with seven response options (1 = no, 2 = yes, but no medical attention was required, 3 = yes, I obtained treatment from my family doctor, 4 = yes, I obtained treatment at a hospital policlinic, 5 = yes, I was hospitalized for 1 night through 2 weeks, 6 = yes, I was hospitalized longer dan 2 week; 7 = yes, I was admitted to the intensive care unit). We collapsed scores into two categories, with scores 1–3 considered as indicating no injury and scores 4–7 indicating physical injury.

#### Hospital Anxiety and Depression Scale (HADS)

The seven item HADS depression scale (HADS-D), part of the 14-item HADS [30] was used to assess depressive symptoms. It instructs respondents to rate their experience of different symptoms (e.g., “I feel as if I am slowed down”) on 4-point scales (scored 0 through 3), with different anchors. The HADS-D showed good psychometric properties, with scores ≥8 pointing at clinically relevant depression [31]. Cronbach’s alpha in the current study was .91.

#### Traumatic Grief Inventory-Self-Report-Plus (TGI-SR+)

The 22 item TGI-SR+ was used to assess grief symptoms connected with the traffic accident. As a slightly extended version of the 18-item TGI-SR [32], the TGI-SR+ was developed to assess symptoms of disordered grief following bereavement [33]. For the present research, items were reworded so that they referred to the changes in a person’s circumstances or experiences caused by the accident. For instance, the item “I found myself longing or yearning for the person who died” was changed into “I found myself longing or yearning for how my life was before the accident”. The frequency of reactions was rated on a scale from 1 = never to 5 = always. In this study, the scale’s Cronbach’s alpha was .97.

#### Posttraumatic Stress Disorder Checklist for DSM-5 (PCL-5)

The 20-item PCL-5 was used to assess PTS symptoms [34, 35]. People rated how often they experienced each symptom in the past month on 5-point scales ranging from 0 = not at all, through 4 = extremely. The instruction and items referred to “the accident” as index event. A provisional DSM-5 [36] based PTSD diagnosis can be made by treating each item rated as ≥2 (moderately) as a symptom endorsed, then following the DSM-5 diagnostic rule which requires at least one criterion B item (questions 1–5), one criterion C item (questions 6–7), two criterion D items (questions 8–14), and two criterion E items (questions 15–20). Cronbach’s alpha of the complete PCL-5 in the current sample was .94.

#### Work and Social Adjustment Scale (WSAS)

The 5-item WSAS was used to assess participant’s perspectives on the degree to which the accident caused functional impairment in the areas of work, home management, social and private leisure activities, and social relations. Items were rated on 9-point scales with anchors 0 = not at all, to 8 = very severely impaired. The original English [37] and Dutch [38] versions have adequate psychometric properties. Cronbach’s alpha in our sample was .96.

#### General Self-Efficacy Scale (GSES)

The GSES, developed by Schwarzer and Jerusalem [39] was used to assess generalized self-efficacy. Participants were instructed to rate its ten items (e.g., “I am confident that I could deal efficiently with unexpected events”) on 4-point scales ranging from 1 = not at all true to 4 = exactly true, with the mean item score used as index of general self-efficacy. Its psychometric properties have been found to be adequate (e.g., [40]). Cronbach’s alpha in our sample was .90.

#### Rumination Questionnaire

The Rumination Questionnaire (RQ) is a 6-item measure assessing trauma rumination [25]. It assesses the frequency of ruminative thoughts associated with a specific traumatic event (e.g., “Do you think about how things could have been different if you had acted differently?”) during the preceding week. Items are scored on 3-point scales (0 = not at all; 1 = somewhat; 2 = very often). Preliminary research supported psychometric properties of the Dutch version [41]. In the current sample the RQ’s alpha was .87.

#### Difficulties in Emotion Regulation Scale-16 (DERS-16)

The DERS-16 is a 16-item version of the 36-item DERS [20], developed by Bjureberg et al. [42] as a tool to assess different dimensions of emotion regulation difficulties, including nonacceptance of negative emotions and inability to engage in goal-directed behaviors when distressed. Respondents rate the extent to which each item applies to them on 5-point scales, ranging from 1 = almost never to 5 = almost always. We used the scale’s total score rather than its subscale scores because evidence shows that the general tendency to regulate emotions (in)flexibly is more relevant to PTS symptomatology than specific forms of emotion dysregulation [23]. Cronbach’s alpha of the total scale in the current sample was .94.

### Statistical analyses

To achieve our first aim, LCA was performed using Mplus 8 [43]. To keep the estimated number of parameters in proportion to the sample size, we used a selection of items from the PTS and grief scales. Specifically, seven items from the PCL-5 were selected as indicators of PTS; considering the DSM-5 symptom clusters, we included two items representing intrusion symptoms, two items representing avoidance, another two items representing negative alterations in cognitions and mood, and one item representing alterations in arousal and reactivity. In selecting items, we considered items of the 5-item Primary Care PTSD Screen for DSM-5 [44]. In addition, ten items from the TGI-SR+ were selected, resembling putative markers of disordered bereavement-related grief as defined in the forthcoming DSM-5-TR [45]. Finally, all seven depression items from the HADS were included. We dichotomized PTS items by considering scores 0 (not at all) and 1 (a little bit) as symptom absent and scores 2 (moderately), 3 (quite a bit), and 4 (extremely) as symptom present. Grief items were dichotomized by considering scores of 1 (never) and 2 (rarely) as item not endorsed, and scores 3 (sometimes), 4 (often), and 5 (always) as item endorsed. For the depression items, the two lowest scores and two highest scores (with varying labels) were considered as symptom absent vs. present, respectively. **Table 1** shows the abbreviated content of all items included in the analyses.

**Table 1:** Abbreviated content of items included in the latent class analysis and probability of item endorsement in the three class solution (N = 328).

Abbreviated content of items	Overall symptom frequency		Class 1: No symptoms class (n = 194), 59.1%		Class 2: Moderate PTS and grief class (n = 76), 23.1%		Class 3: Severe symptoms class (n = 58), 17.7%		Differences in continuous symptom scores between classes
Posttraumatic Stress	%	N	Probability	SE	Probability	SE	Probability	SE	
1 Intrusive memories	25	84	0.041	0.016	0.445	0.060	0.718	0.061	C1<C2<C3
2 Disturbing dreams	10	34	0.006	0.006	0.116	0.038	0.412	0.069	C1<C2<C3
3 Avoiding memories, thoughts	20	67	0.049	0.017	0.318	0.057	0.568	0.072	C1<C2<C3
4 Avoiding external reminders	22	73	0.069	0.020	0.384	0.060	0.516	0.071	C1<C2<C3
5 Blaming yourself, others	28	94	0.150	0.027	0.425	0.059	0.557	0.067	C1<C2 = C3
6 Distant from others	17	57	0.005	0.005	0.126	0.041	0.798	0.056	C1<C2<C3
7 “Superalert”, watchful	36	119	0.160	0.028	0.599	0.059	0.724	0.059	C1<C2<C3
Depression									
1 Not enjoying things	16	53	0.035	0.015	0.056	0.030	0.722	0.063	C1 = C2<C3
2 Cannot laugh/see sunny side	11	38	0.006	0.006	0.000	0.000	0.636	0.068	C1 = C2<C3
3 Not feel cheerful	15	50	0.066	0.018	0.068	0.030	0.551	0.071	C1 = C2<C3
4 Feel slowed down	23	76	0.064	0.019	0.153	0.049	0.893	0.041	C1<C2<C3
5 Lost interest in appearance	14	49	0.062	0.018	0.066	0.030	0.551	0.069	C1 = C2<C3
6 Don’t look forward to things	12	42	0.016	0.009	0.018	0.019	0.647	0.066	C1 = C2<C3
7 Don’t enjoy book/radio/tv	22	73	0.122	0.025	0.149	0.045	0.654	0.067	C1 = C2<C3
Grief									
1 Yearning	27	89	0.050	0.017	0.368	0.065	0.878	0.046	C1<C2<C3
2 Preoccupation	26	87	0.048	0.017	0.469	0.062	0.717	0.061	C1<C2<C3
3 Identity confusion	20	67	0.000	0.000	0.233	0.056	0.843	0.050	C1<C2<C3
4 Disbelief	24	79	0.039	0.015	0.433	0.063	0.654	0.063	C1<C2<C3
5 Avoidance	17	57	0.024	0.012	0.339	0.058	0.451	0.069	C1<C2<C3
6 Emotional pain	25	84	0.000	0.000	0.436	0.063	0.867	0.050	C1<C2<C3
7 Difficulties moving on	17	56	0.000	0.000	0.182	0.051	0.723	0.061	C1<C2<C3
8 Numbness	15	48	0.000	0.000	0.178	0.046	0.590	0.068	C1<C2<C3
9 Meaninglessness	12	40	0.000	0.000	0.038	0.023	0.638	0.069	C1<C2<C3
10 Loneliness	19	64	0.000	0.000	0.217	0.057	0.813	0.052	C1<C2<C3

Next, we evaluated models with one up to six classes, to determine the optimal number of latent classes in the dataset. Model selection was based on statistical indices, interpretability, and parsimony [46]. The fit of successive models was evaluated using the following indices: the Bayesian information criterion (BIC), sample-size adjusted BIC (SA-BIC), and Akaike’s information criterion (AIC) with lower values indicating better fit, the entropy R^2^, with values >.80 considered acceptable, and the bootstrap likelihood ratio test (BLRt) and Vuong, Lo, Mendel, Rubin likelihood ratio test (VLRt) of which p-values < 0.05 indicate a significant improvement of the fit of a model relative to the model with one less class. Furthermore, class solutions that included very few participants were not retained because these might yield computational difficulties (e.g., inaccurate estimates of class sizes) and elevated risk for Type 2 error when examining correlates of these classes. Lastly, parsimonious solutions were preferred over more complex solutions. Next, for exploratory reasons, we examined differences between classes in terms of continuous scores on the items included as indicators. To this end, we performed a series of analyses of variance (ANOVAs) in SPSS (Version 25) testing differences in continuous (i.e. non-dichotomized) symptom scores between emerging classes.

To address our second aim, we used the three-step approach implemented in Mplus to examine differences between classes in terms of functional impairment (WSAS), as well as total scores on the PCL-5, the summed scores of items tapping the B-, C-, D-, and E-clusters, the TGI-SR+, and the HADS depression scale. Chi square testing examined if classes different in terms of percentages of people meeting vs. not meeting criteria for probable PTSD and clinically relevant depression. We also used the three-step approach to examine if class membership differed as a function of sociodemographic variables and characteristics of the traffic accident (aim 3) and scores on indices of self-efficacy, difficulties in emotion regulation, and trauma-rumination (aim 4). This was followed up by a regression analysis (using the three-step approach) in which sociodemographic, accident-related, and psychological variables emerging as significant predictors of class-membership in the univariate analyses were simultaneously entered as independent variables.

## Results

### Descriptive data and confirmatory factor analysis

**Table 2** summarizes sample characteristics. Participant’s mean age was 32.5 (SD = 17.4) years; most were female and most were highly educated. Time since the accident was seven years on average and ranged from several weeks to 68 years. Regarding the transportation type, 47% were in a car or on a motorcycle during the accident and the remaining participants used a variety of transportation types (e.g., biking, walking). Two thirds were driving the transportation vehicle. The experienced threat to life averaged M = 3.44, on a 1 to 7 point scale. With respect to injury severity, almost 20% were categorized as physically injured. Using the diagnostic algorithm for PTSD as per DSM-5, n = 44 (13.4%) met criteria for probable PTSD based on their PCL-5 scores. Further, n = 70 (21.3%) scored ≥8 on the HADS suggesting clinically relevant levels of depression [29].

**Table 2:** Characteristics of participants by class in the three class solution (N = 328).

	Total group	Class 1: No symptoms class (n = 194), 59.1%	Class 2: Moderate PTS and grief class (n = 76), 23.1%	Class 3: Severe symptoms class (n = 58), 17.7%
**Sociodemographic background variables**				
Gender, *N (%)*				
Male	109 (33.2)	62 (31.9)	26 (34.2)	21 (36.2)
Female	219 (66.8)	132 (68.1)	50 (65.8)	37 (63.8)
Age, *M (SD)*, *range*	32.5 (17.4), 18–87	26.2 (13.2), 18–87	38.3 (19.5), 18–80	46.1 (17.0), 18–79
Education, *N (%)*				
Lower than college/university	208 (63.4)	138 (71.1)	36 (47.3)	34 (58.6)
College/university	120 (36.6)	56 (28.9)	40 (52.7)	24 (41.4)
**Characteristics of the traffic accident**				
Months since accident, M (SD); Median (IQR) ^a^	79.1 (107.4); 48 (24–91)	62.8 (52.1); 48 (25–87.5)	88.6 (150.6); 32 (18–75)	120.1 (155.3); 56 (245.2–122)
Type of transportation during the accident, *N (%)*				
Car/motorcycle	155 (47.3)	93 (47.9)	32 (42.1)	30 (51.7)
Other	173 (52.7)	101 (52.1)	44 (57.9)	28 (48.3)
Were you driver of the transportation vehicle, N (%) ^b^				
No	96 (30.2)	70 (36.5)	19 (26.4)	7 (13.0)
Yes	222 (69.8)	122 (63.5)	53 (73.6)	47 (87.0)
Perceived threat to life (range 1–7), M (SD)	3.44 (2.18)	2.89 (1.62)	4.07 (2.40)	4.45 (2.87)
Were you physically injured in the accident?				
No injury	263 (80.2)	178 (91.8)	53 (69.7)	32 (55.2)
Physical injury	65 (19.8)	16 (8.2)	23 (30.3)	15 (44.8)

Note.

^a^ There were missing values for this variable (total group n = 309, Class 1, n = 182, Class 2, n = 71, Class 3, n = 56.

^b^ There were missing values for this variable, total group n = 318.

For exploratory reasons, we examined if the symptoms of PTS, depression, and grief that were included in the LCAs represented distinguishable dimensions. To this end, we used confirmatory factor analysis (CFA), to compare the fit of a one-factor model (with all items loading on one dimension) with the fit of a three-factor model (with items forming three distinct factors). CFA was performed using Mplus, with dichotomized items scores (similar to the LCA) and employing the robust weighted least squares estimation method (WLSMV). Findings showed that the one-factor model fit the data (CFI = 0.964, TLI = 0.960, RMSEA = 0.069, SRSR = 0.101). The three-factor model, however, had even better fit indices (CFI = 0.974, TLI = 0.972, RMSEA = 0.059, SRSR = 0.085). The Chi-square difference test (calculated using the DIFFTEST command in Mplus) supported that the three-factor model fit better (Δχ^2^ = 49.12 (3), p < .001).

### Latent classes of PTSD, depression, and grief symptoms

**Fig 1:**
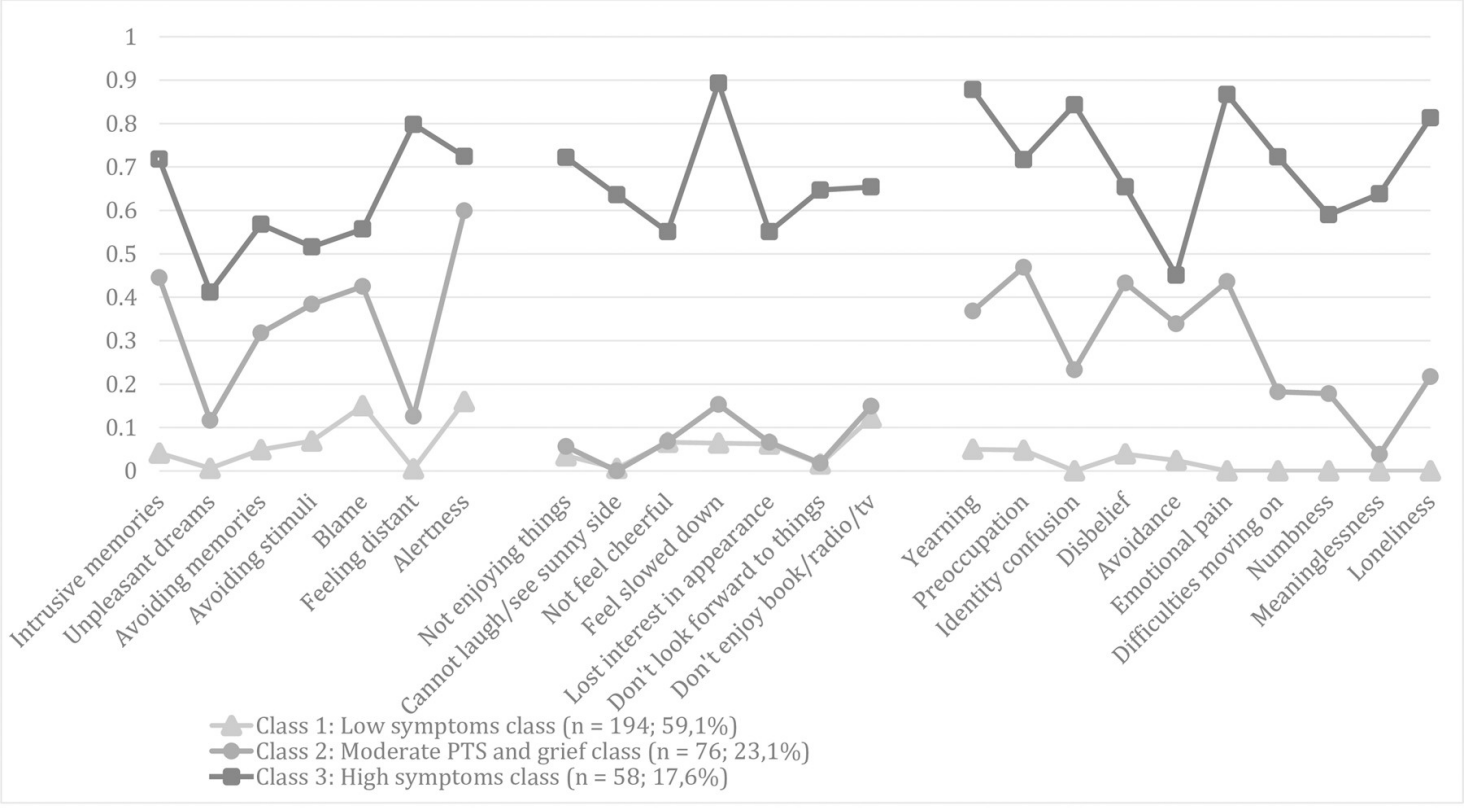
Probabilities of symptom endorsement for each latent class in the three class solution.

The goodness of fit indices for the one class through six class solutions are shown in **Table 3**. Fit indices did not clearly point at one best fitting model: the BIC was lowest for the four class solution, but the VLRt-value indicated this model was not a significant improvement of the three class model. The VLRt values of the three class model indicated that this model fit better than the two class model. AIC and SA-BIC values continued to decrease gradually when the number of classes increased, but the magnitude of reductions in these values was small beyond the four class solution. On inspection of the three and four class solutions, we saw that, of all 194 members of class 1 in the three class solution, 193 (99.4%) were also in class 1 in the four class solution; of all 76 members of the second class in the three class solution, 61 (80.2%) were included in a second class in the four class solution; of all 58 members of the third class of the three class solution, 25 (43.1%) were members of class 3 and 33 (59.9%) of class 4 of the four class solution. Inspection of item probabilities indicated that the third class in the three class solution (evidencing a high probability of most symptoms) was split in two, almost equally sized classes (evidencing moderately vs. high probability of most symptoms) in the four class solution. Considering parsimony and interpretability, the three class solution was retained. **Fig 1** shows symptom probabilities and **Table 1** shows symptom frequencies and probabilities of item endorsement across the three classes.

**Table 3:** Goodness-of-fit indices for latent class models (N = 328).

Model	Log Likelihood	AIC	BIC	SA-BIC	Entropy	BLRt p value	VLRt p-value	Sample size by class based on most likely membership
1 class	-3853.29	7754.49	7845.53	7769.40				328
2 class	-2841.56	5781.13	5966.99	5811.56	0.97	< 0.001	< 0.001	76/252
3 class	-2675.18	5498.37	5779.05	5544.32	0.94	< 0.001	< 0.001	58/76/194
4 class	-2581.72	5361.46	5736.96	5422.94	0.94	< 0.001	0.065	33/36/62/197
5 class	-2541.49	5330.99	5801.32	5408.00	0.94	< 0.001	0.541	23/24/36/149/196
6 class	-2503.48	5304.96	5870.12	5397.50	0.94	< 0.001	0.285	16/24/27/35/44/182

Note. AIC = Akaike information criterion. BIC = Bayesian information criterion. BLRt = Bootstrap Likelihood Ratio test. SA-BIC = Sample-size adjusted Bayesian information criterion. VLRt = Vuong-Lo-Mendell-Rubin likelihood ratio test.

Consistent with prior LCAs (e.g., [14]), values ≤0.15 were considered as representing a low probability that the symptom was present, values between 0.15 and 0.59 a moderate probability, and values ≥0.60 a high probability that the symptom was present. Accordingly, Class 1 included 194 individuals (59.1%) with low endorsement of all but one symptoms (i.e. “alertness”, with a probability of 0.16 that was very close to the ≤0.15 threshold). This class was named the "no symptoms class". Class 2 included 76 (23.1%) individuals demonstrating moderate endorsement of five PTS, one depression, and all grief symptoms. This class was named the “moderate PTS and grief class”. Class 3 included 58 individuals (17.6%) evidencing high endorsement of three PTS, five depression, and eight grief items and moderate endorsement of all other items. This class was named the “severe symptom class”.

### Differences in continuous symptom scores between classes

We used Welch’s ANOVAs (because the assumption of homogeneity of variance was violated) to compare the continuous scores of all items included in the LCA. F-values were statistically significant for all 24 item scores (all F’s > 33.10, all p’s < 0.001). Post-hoc testing showed that, for six PTS symptoms, scores were lowest in the no symptoms class, significantly higher in the moderate PTS and grief class, and highest in the severe symptoms class (i.e. Class 1 < Class 2 < Class 3). For item 5 (blaming yourself, others), the moderate PTS and grief class and severe symptoms class scored higher than the no symptoms class, but did not differ from each other (i.e. Class 1 < Class 2 = Class 3). For depression, item scores were ordered Class 1 < Class 2 < Class 3 for item 4 (feeling slowed down) and were ordered Class 1 = Class 2 < Class 3 for all other items. For grief, all ten item scores differed significantly between groups with scores ordered Class 1 < Class 2 < Class 3 for all items (see Table 1).

### Differences in total symptom scores and functional impairment between classes

**Table 4** shows scores on the indices of functional impairment, PTS, depression, and grief in the total sample and the classes. **S1 Table** summarizes outcomes of the distinct regression analyses with class membership consecutively regressed on all these indices, using the three-step approach. All pairwise comparisons were statistically significant at the p < .05 level such that scores were lowest in Class 1, higher in Class 2, and highest in Class 3. When employing a Bonferroni correction, using a more conservative threshold of p < 0.05/24 (i.e., the number of pairwise comparisons) = .002, the no symptoms class and moderate PTS and grief class did not differ in terms of depression severity and the moderate PTS and grief class and severe symptoms class did not differ in terms of PTS avoidance.

**Table 4:** Mean scores on indices of emotional distress and functioning by class in the three class solution (N = 328).

Emotional distress and functioning (N = 328)				
	Total group	Class 1: No symptoms class (n = 194), 59.1%	Class 2: Moderate PTS and grief class (n = 76), 23.1%	Class 3: Severe symptoms class (n = 58), 17.7%
PTS total score (PCL-5), M (SD)	15.11 (15.10)	6.17 (4.95)	19.14 (9.06)	39.74 (14.37)
PTS B cluster items, M (SD)	3.35 (4.13)	1.18 (1.55)	4.72 (3.48)	8.83 (4.98)
PTS C cluster items, M (SD)	1.48 (1.96)	0.58 (0.99)	2.40 (2.01)	3.31 (2.47)
PTS D cluster items, M (SD)	5.16 (5.81)	1.97 (2.19)	5.88 (3.55)	14.87 (5.49)
PTS E cluster items, M (SD)	5.10 (5.08)	2.42 (2.60)	6.13 (3.89)	12.72 (4.49)
Grief (TGI-SR-+), M (SD)	36.40 (18.70)	24.94 (4.17)	41.28 (10.35)	68.36 (17.02)
Depression (HADS-D), M (SD)	4.46 (4.60)	2.55 (2.58)	3.31 (2.49)	12.36 (3.64)
Functional impairment (WSAS), M (SD)	7.56 (10.69)	2.04 (4.05)	8.84 (8.88)	24.18 (10.34)
Psychological variables (N = 296)				
	Total group	Class 1: No symptoms class (n = 179), 60.5%	Class 2: Moderate PTS and grief class (n = 68), 22.9%	Class 3: Severe symptoms class (n = 49), 16.5%
Generalized self-efficacy (GSES), M (SD)	3.00 (0.52)	3.09 (0.46)	3.00 (0.48)	2.65 (0.67)
Difficulties in emotion regulation (DERS-16), M (SD)	35.16 (13.66)	33.57 (12.48)	32.79 (12.95)	44.26 (15.28)
Trauma rumination (RQ), M (SD)	2.32 (3.00)	0.81 (1.41)	3.48 (2.77)	6.22 (3.37)

*Note*. DERS-16 = Difficulties in Emotion Regulation Scale-16. GSES = General Self-Efficacy Scale. HADS = Hospital Anxiety and Depression Scale. PCL-5 = Posttraumatic Stress Disorder Checklist for DSM-5. PTS = Posttraumatic Stress. RQ = Rumination Questionnaire. TGI-SR+ = Traumatic Grief Inventory-Self-Report-Plus. WSAS = Work and Social Adjustment Scale.

### Differences in probable PTSD and depression “caseness” between classes

The percentage of participants meeting vs. not meeting criteria for probable PTSD, based on the PCL-5, were 0.5% vs. 99.5% in Class 1, 14.5% vs. 85.5% in Class 2, and 55.2% vs. 44.8% in Class 3. Percentages differed significantly between classes (χ^2^ (2, N = 328) = 114.94, p < 0.001). Bonferroni adjusted z-tests for column proportions showed that the percentage was significantly higher in Class 2 compared to Class 1, and significantly higher in Class 3 compared to Class 1 and Class 2. The percentage of participants meeting vs. not meeting criteria for clinically relevant depression, based on the HADS cutoff of ≥8 were 5.2% vs. 94.8% in Class 1, 6.6% vs. 93.3% in Class 2, and 94.8% vs. 5.2% in Class 3. Percentages differed significantly between classes (χ^2^ (2), N = 328) = 226.72, p < 0.001). Bonferroni adjusted z-tests for column proportions showed that the percentage was significantly higher in Class 3 compared to Class 1 and Class 2, with no differences between these latter classes.

### Differences in sociodemographic and accident related variables between classes

**Table 2** summarizes sociodemographic and accident related variables across classes. Distinct regression analyses (with the three-step approach) were carried out in which class membership was consecutively regressed on these variables to statistically test if classes differed in terms of these variables. Outcomes are summarized in **S2 Table**. Classes did not differ in terms of gender. Age differed between classes, such that people in the no symptoms class (Class 1) were younger than those in the moderate PTS and grief class (Class 2) who, in turn, were younger than those in the severe symptoms class (Class 3). As for education, participants with higher education had an elevated chance of inclusion in Class 2 compared to Class 1. Looking at accident characteristics, classes differed by time since the accident, such that for those in Class 3, the accident was significantly longer ago, than people in Class 1. No class differences emerged on (dichotomized) transportation type. Being a driver differed between Class 3 and Class 1, with people in Class 3 being more often the driver. Both the perceived threat to life and having experienced physical injury increased the probability of being included in Class 2 vs. Class 1 and Class 3 vs. Class 1, but did not differ between Classes 2 and 3.

### Differences in psychological variables between classes

Table 4 shows scores on indices of self-efficacy, difficulties in emotion regulation, and trauma rumination in the three classes. Three consecutive regression analyses, using the three-step approach, tested differences between classes in terms of these variables. Outcomes are summarized in **S3 Table**. Self-efficacy was significantly lower in Class 3 compared to Class 1 and Class 2 but did not differ between Class 1 and 2. Likewise, difficulties in emotion regulation were higher in Class 3 than Class 1 and Class 2, but did not differ between Class 1 and 2. Trauma rumination differentiated between all classes and was ordered Class 1 < Class 2 < Class 3.

### Regression analyses included all significant univariate correlates

Next, we performed a regression using the three-step approach including age, education, time since the accident, being a driver of the vehicle involved in the accident, perceived threat to life, and dichotomized physical injury, plus the three psychological variables. Outcomes, summarized in **S4 Table**, showed that lower self-efficacy and higher trauma rumination (but none of the other variables) differentiated Class 2 from Class 1. Apart from age and being a driver of the vehicle, all three psychological variables differentiated Class 3 from Class 1. Finally, age as well as difficulties in emotion regulation and trauma rumination differentiated Class 3 from Class 2. When employing a Bonferroni correction, using a more conservative threshold of p < 0.05/27 (i.e., the number of pairwise comparisons) = .002, trauma rumination was the single variable differentiating Class 2 from Class 1; self-efficacy, difficulties with emotion regulation, and trauma rumination differentiated Class 3 from Class 1; and difficulties with emotion regulation and trauma rumination differentiated Class 3 from Class 2.

## Discussion

In this research, we examined whether meaningful subgroups of victims of non-fatal traffic accidents could be identified based on their scores on measures assessing PTS symptoms, depression symptoms, and grief reactions. To our knowledge, this study is the first to consider grief reactions connected with potentially traumatic events that do not include the death of a loved one. Based on LCA, we identified three classes: a class with low probabilities of endorsing symptoms across all three clusters, a second class evidencing moderate probabilities of endorsing symptoms of PTS and grief, and a third class characterized by high probabilities of endorsing the majority of symptoms from all three clusters. The first class was the largest, which is not unexpected given that the majority of people is resilient in the face of traumatic events [47]. Notably, we did not find a class with predominantly elevated PTS; instead, in the three classes, the levels of PTS and grief seemed to co-occur at similar, gradually increasing levels of severity. Findings indicate that victims of non-fatal accidents afflicted with moderate to severe distress experience a sense of ongoing threat—connected with the threat experienced during the event (cf. [26])—as well as grief about losses incurred as a result of the event. In Class 3, odds of traumatic stress, depression, and grief were more pronounced. This indicates that those with the most pervasive symptoms are likely to experience a combination of symptoms, which is generally consistent with evidence that severe PTSD symptomatology and depression often co-occur after trauma [13] and loss [48].

A second main finding was that classes differed in terms of functional impairment and mental health symptoms. That is, summed scores on the measures of PTS, depression, and grief and functional impairment were significantly higher in the severe symptoms class (Class 3) compared to the no symptoms class (Class 1) and moderate PTS and grief class (Class 2); this provides confirmation that emotional suffering was greatest in that group. Notably, members of Class 2 scored worse on these outcomes (except depression) than members of Class 1, even though the large majority of participants in Class 2 did not meet criteria for probable PTSD and clinically relevant depression. Findings suggest that people experiencing moderate distress—many of whom do not pass the threshold for a full-blown disorder—are still afflicted by significant distress and impairment. This accords with prior findings that subclinical psychopathology may yield substantial distress [8]. It suggests that an exclusive reliance on dichotomous classification to provide people with therapeutic interventions is problematic because it may deprive a sizeable group of psychological help, such as exposure-based writing therapy that has shown promising results [9].

Concerning our third aim, to identify sociodemographic variables and characteristics of the accident associated with class-membership, we found that age and education differed between classes. Age was ordered as Class 1 < Class 2 < Class 3; participants with higher education had a greater likelihood of membership of the moderate PTS and grief class (Class 2) than Class 1 and Class 3. Considering characteristics of the accident, we found that the time since the accident was significantly longer in Class 3 compared to Class 1. This finding is probably due to the fact that we used a self-selected sample; that is, people with more severe symptomatology may consider the accident more personally relevant and therefore may be more likely to participate longer after the event. In addition, this finding may reflect that Class 3 includes relatively many people with chronic PTSD symptomatology which is usually more severe. Being a driver of the vehicle involved in the accident increased the chance of membership of Class 3 vs. Class 1. This may be connected with stronger feelings of guilt and self-blame among those driving the vehicle. Greater perceived threat to life and physical injury distinguished both the moderate PTS and grief and severe symptoms classes from the no symptoms class, but did not differentiate between the moderate and severe classes (Class 1 < Class 2 = Class 3). This indicates that both these variables are not very critical to understanding who develops intermediate PTS and grief vs. more severe symptoms of PTS, depression, and grief. That a greater threat to life during the accident was associated with a greater likelihood of inclusion in Class 2 and Class 3 is consistent with prior evidence that this variable is a strong predictor of PTSD following different traumatic events [49], including traffic accidents [2]. Reliving and trying to avoid threat are at the heart of traumatic stress and, therefore, it seems logical that traumatic stress is more intense in the face of more intense threat to one’s own life. From a neurobiological perspective, the linkage between acute life-threatening situations and intense PTSD symptomatology may stem from adrenergic arousal that is involved in the formation as well as the recall of traumatic memories (cf. [49]).

The fourth aim was to examine the role of transdiagnostic psychological mechanisms in distinguishing symptom patterns. Univariate analyses showed that self-efficacy was lower and difficulties in emotion regulation were higher in the severe symptoms class (Class 3) compared to the no symptoms class and the moderate PTS and grief class (Class 1 and Class 2), but did not differentiate between Class 1 and Class 2. Trauma rumination differentiated between all classes and was highest in Class 3, lower in Class 2, and lowest in Class 1. In a final regression analysis, including all significant univariate predictors of class membership, lower self-efficacy and higher trauma rumination differentiated Class 2 from Class 1. Self-efficacy, difficulties with emotion regulation, and trauma rumination differentiated Class 3 from Class 1. And difficulties with emotion regulation and trauma rumination differentiated Class 3 from Class 2. This sheds some light on potential underlying mechanisms of the most severe emotional responses to traffic accidents. That is, self-efficacy and emotion regulation difficulties are seemingly less important in increasing the chance of membership of Class 2 (vs. Class 1); self-efficacy is less important in elevating chances of membership of Class 3 (instead of Class 2). Gradually increasing trauma rumination coincides with gradually increasing chances of experiencing more severe symptoms overall. This latter finding is broadly consistent with prior studies pointing at the detrimental role of this thinking style in traumatic stress and depression following trauma [1, 24, 25] and loss [50]. The negative impact of rumination may be due to the fact that repetitively going over the cause and consequences of a traumatic event blocks emotional processing, thereby maintaining PTS. Moreover, rumination maintains an inward focus that impairs engagement in constructive action fostering adjustment, thereby perpetuating feelings of traumatic stress as well as depression and grief [51, 52].

The present findings should be considered in light of several potential limitations. First, the use of a cross-sectional design was suitable for our goal to identify subgroups of victims based on profiles of PTS, depression, and grief. However, it precludes conclusions about the stability of these profiles and about whether the correlates of classes that we considered are prospective predictors of symptom trajectories. Second, we recruited participants at universities (alongside other channels) and all participants were self-selected. Therefore, students were overrepresented and the sample likely does not adequately represent the entire target population of victims of non-fatal accidents. Further, that members of the most severe class (Class 3) were oldest suggests that some of the observations in this study are a consequence of PTS symptom severity and chronicity, which may hinder the generalizability of findings. Nevertheless, some findings, including those concerning prevalence rates of probable PTSD and the key role of perceived threat to life and trauma rumination in explaining symptom profiles are compatible with prior work (cf., [1, 49]). Third, and relatedly, while we sought to examine the impact of a single category of events—traffic accidents—there is obviously substantial heterogeneity in the nature of these events, in terms of potential lethality, other people involved (other victims, people responsible), and time elapsed since the event. Therefore, caution should be applied in translating outcomes to the experience of victims of specific types of accidents (e.g., a mild bicycle crash vs. motor vehicle accident with multiple casualties). Fourth, although LCA was an appropriate approach to achieve our study aims, LCA is not without limitations (see [53]). For instance, limitations of the sample size may cause particular subgroups to be overlooked and how emerging subgroups are characterized and described is, of course, limited by the indicators that are used to identify groups in the first place. Moreover, subgroups identified within one sample do not necessarily represent actual subgroups in the population [53]. Therefore, caution should be applied in generalizing our findings and more research is needed, examining if similar subgroups emerge in other, large samples, and when different types of indicators for PTS, depression, and grief are used.

## Conclusion

Notwithstanding these considerations, this study enhances our understanding of the heterogeneity in the presentation of PTS and related symptoms, among traffic accident victims. The findings bolster prior evidence that the majority of these people develop few, if any, emotional complaints [2], but that still one of six people shows patterns of moderate mental health symptoms, and an similarly sized group is afflicted with severe mental health symptoms. The finding of higher distress among those people involved in more severe incidents aligns with prior research [2] and provides further evidence that these people are candidates for early preventative psychological care. One key finding of this study was that PTS and grief co-occurred across the classes. This indicates that feelings of grief (e.g., yearning for life as it was before the accident, difficulties accepting changes caused by the accident) are part of the emotional experience of victims of traffic accidents as they continue their lives after the event. This has clinical relevance, e.g., victims may find solace when caregivers acknowledge and give the right words to their emotional experiences. Moreover, coming to terms with involvement in a traffic accident not only concerns the elaboration and integration of memories of the circumstances of the accident [26], but also requires some degree of emotionally processing the life-changes and losses caused by the event.

Among those experiencing severe grief reactions, caregivers might foster grief processing by helping people to confront losses and life-changes, articulate the meanings and implications of the event, and give life new meaning by adjusting life goals and activities in line with this new situation. Findings of this study also suggest the importance of addressing trauma rumination in treating emotional problems following traffic accidents. Interventions useful for this aim include using attention training to cultivate people’s awareness of unhelpful repetitive thinking and altering interpretations about the usefulness of rumination [51, 54]. Given that rumination often serves to avoid the pain connected with traumatic memories, fostering emotional processing of these memories helps to reduce rumination. Indeed, randomized controlled trials have shown that exposure to avoided aspects of loss and trauma reduced rumination [54]. When depressive symptoms dominate the clinical picture, trauma-focused interventions may be more appropriate, considering that these interventions alleviate both traumatic stress and depression symptoms [55].

## Supporting information

S1 Data (SAV)

S1 Table Summary of distinct regression analyses with symptom levels and functional impairment predicting class membership. (DOCX)

S2 Table Summary of distinct regression analyses with sociodemographic and loss-related variables predicting class membership. (DOCX)

S3 Table Summary of distinct regression analyses with self-efficacy, difficulties in emotion regulation, and rumination predicting class membership. (DOCX)

S4 Table Summary of regression analyses with sociodemographic and loss-related variables, self-efficacy, difficulties in emotion regulation, and trauma rumination predicting class membership. (DOCX)

S1 Fig Flow-chart of participants. (DOCX)

## References

[1] Ehring T, Ehlers A, Glucksman E. Do cognitive models help in predicting the severity of posttraumatic stress disorder, phobia, and depression after motor vehicle accidents? A prospective longitudinal study. J Consult Clin Psychol. 2008;76(2):219–30. doi: 10.1037/0022-006X.76.2.219 18377119 PMC2672053

[2] Heron-Delaney M, Kenardy J, Charlton E, Matsuoka Y. A systematic review of predictors of posttraumatic stress disorder (PTSD) for adult road traffic crash survivors. Injury. 2013;44(11):1413–22. doi: 10.1016/j.injury.2013.07.011 23916902

[3] Harris DL. Non-death loss and grief: Context and clinical implications. New York: Routledge. 2019

[4] Papa A, Lancaster NG, Kahler J. Commonalities in grief responding across bereavement and non-bereavement losses. J Affect Disord. 2014;161:136–43. doi: 10.1016/j.jad.2014.03.018 24751321

[5] Clark DM. Developing new treatments: on the interplay between theories, experimental science and clinical innovation. Behav Res Ther. 2004;42(9):1089–104. doi: 10.1016/j.brat.2004.05.002 15325903

[6] Collins L.M, Lanza ST. Latent class and latent transition analysis: With applications in the social, behavioral and Health Sciences. New York: Wiley. 2010.

[7] Howard MC, Hoffman ME. Variable-centered, person-centered, and person-specific approaches: Where theory meets the method. *Organizational Res Methods*. 2018;21(4):846–876. 10.1177/1094428117744021

[8] Brancu M, Mann-Wrobel M, Beckham JC, Wagner HR, Elliott A, Robbins AT, et al. Subthreshold posttraumatic stress disorder: A meta-analytic review of DSM-IV prevalence and a proposed DSM-5 approach to measurement. Psychol Trauma. 2016;8(2):222–32. doi: 10.1037/tra0000078 26390108

[9] Dawson RL, Calear AL, McCallum SM, McKenna S, Nixon RDV, O’Kearney R. Exposure-based writing therapies for subthreshold and clinical posttraumatic stress disorder: A systematic review and meta-analysis. J Trauma Stress. 2021;34(1):81–91. doi: 10.1002/jts.22596 33043507

[10] Djelantik AAAMJ Robinaugh DJ, Kleber RJ Smid GE, Boelen PA. Symptomatology following loss and trauma: Latent class and network analyses of prolonged grief disorder, posttraumatic stress disorder, and depression in a treatment-seeking trauma-exposed sample. Depress Anxiety. 2020;37(1):26–34. doi: 10.1002/da.22880 30724427 PMC7004006

[11] Lenferink LIM, de Keijser J, Smid GE, Djelantik AAAMJ, Boelen PA. Prolonged grief, depression, and posttraumatic stress in disaster-bereaved individuals: latent class analysis. Eur J Psychotraumatol. 2017;8(1):1298311. doi: 10.1080/20008198.2017.1298311 28451067 PMC5399993

[12] Van Eersel JHW, Taris TW, Boelen PA. Grief reactions, depression, and anxiety following job loss: Patterns and correlates. European Journal of Psychotraumatology. 2021:12(1), 1–13. doi: 10.1080/20008198.2021.2000131 36877469 PMC9754011

[13] Au TM, Dickstein BD, Comer JS, Salters-Pedneault K, Litz BT. Co-occurring posttraumatic stress and depression symptoms after sexual assault: a latent profile analysis. J Affect Disord. 2013;149(1–3):209–16. doi: 10.1016/j.jad.2013.01.026 23489401

[14] Minihan S, Liddell BJ, Byrow Y, Bryant RA, Nickerson A. Patterns and predictors of posttraumatic stress disorder in refugees: A latent class analysis. J Affect Disord. 2018;232:252–9. doi: 10.1016/j.jad.2018.02.010 29499508

[15] Delahanty DL, Raimonde AJ, Spoonster E, Cullado M. Injury severity, prior trauma history, urinary cortisol levels, and acute PTSD in motor vehicle accident victims. J Anxiety Disord. 2003;17(2):149–64. doi: 10.1016/s0887-6185(02)00185-8 12614659

[16] Bandura A. Self-efficacy: The exercise of control. New York: Freeman. 1997.

[17] Bosmans MWG, van der Velden PG. Longitudinal interplay between posttraumatic stress symptoms and coping self-efficacy: A four-wave prospective study. Soc Sci Med. 2015;134:23–9. doi: 10.1016/j.socscimed.2015.04.007 25875423

[18] Nygaard E, Johansen VA, Siqveland J, Hussain A, Heir T. Longitudinal relationship between self-efficacy and posttraumatic stress symptoms 8 years after a violent assault: An autoregressive cross-lagged model. Front Psychol. 2017;8:913. doi: 10.3389/fpsyg.2017.00913 28620334 PMC5452477

[19] Maciejewski PK, Prigerson HG, Mazure CM. Self-efficacy as a mediator between stressful life events and depressive symptoms. Differences based on history of prior depression. Br J Psychiatry. 2000;176:373–8. doi: 10.1192/bjp.176.4.373 10827887

[20] Lacour O, Morina N, Spaaij J, Nickerson A, Schnyder U, von Känel R, et al. Prolonged Grief Disorder among refugees in psychological treatment-association with self-efficacy and emotion regulation. Front Psychiatry. 2020;11:526. doi: 10.3389/fpsyt.2020.00526 32581893 PMC7291948

[21] Gross JJ. Emotion regulation: current status and future prospects. Psychological Inquiry. 2015;26:1–26. 10.1080/1047840X.2014.940781

[22] Gratz KL, Roemer L. Multidimensional assessment of emotion regulation and dysregulation: Development, factor structure, and initial validation of the difficulties in emotion regulation scale. J Psychopathol Behav Assess. 2004;26(1):41–54. 10.1023/B:JOBA.0000007455.08539.94

[23] Seligowski AV, Lee DJ, Bardeen JR, Orcutt HK. Emotion regulation and posttraumatic stress symptoms: a meta-analysis. Cogn Behav Ther. 2015;44(2):87–102. doi: 10.1080/16506073.2014.980753 25421727

[24] Ehlers A, Mayou RA, Bryant B. Psychological predictors of chronic posttraumatic stress disorder after motor vehicle accidents. J Abnorm Psychol. 1998;107(3):508–19. doi: 10.1037//0021-843x.107.3.508 9715585

[25] Murray J, Ehlers A, Mayou RA. Dissociation and post-traumatic stress disorder: two prospective studies of road traffic accident survivors. Br J Psychiatry. 2002;180(4):363–8. doi: 10.1192/bjp.180.4.363 11925361

[26] Ehlers A, Clark DM. A cognitive model of posttraumatic stress disorder. Behav Res Ther. 2000;38(4):319–45. doi: 10.1016/s0005-7967(99)00123-0 10761279

[27] Lenferink LIM, de Keijser J, Eisma MC, Smid GE, Boelen PA. Treatment gap in bereavement care: (Online) bereavement support needs and use after traumatic loss. Clin Psychol Psychother. 2021;28(4):907–16. doi: 10.1002/cpp.2544 33377266 PMC8451936

[28] Lenferink L, de Keijser J, Eisma M, Smid G, Boelen P. Online cognitive-behavioural therapy for traumatically bereaved people: study protocol for a randomised waitlist-controlled trial. BMJ Open. 2020;10(9):e035050. doi: 10.1136/bmjopen-2019-035050 32883723 PMC7473627

[29] Mayou R, Bryant B. Outcome 3 years after a road traffic accident. Psychol Med. 2002;32(4):671–5. doi: 10.1017/s0033291702005470 12102381

[30] Zigmond AS, Snaith RP. The hospital anxiety and depression scale. Acta Psychiatr Scand. 1983;67(6):361–70. doi: 10.1111/j.1600-0447.1983.tb09716.x 6880820

[31] Bjelland I, Dahl AA, Haug TT, Neckelmann D. The validity of the Hospital Anxiety and Depression Scale. J Psychosom Res. 2002;52(2):69–77. doi: 10.1016/s0022-3999(01)00296-3 11832252

[32] Boelen PA, Smid GE. The traumatic grief inventory self-report version (TGI-SR): Introduction and preliminary psychometric evaluation. J Loss Trauma. 2017;22(3):196–212. 10.1080/15325024.2017.1284488

[33] Lenferink L.I.M., Eisma M.C., Smid G.E., de Keijser J., & Boelen P.A. (2022). Valid measurement of DSM-5 persistent complex bereavement disorder and DSM-5-TR and ICD-11 prolonged grief disorder: The Traumatic Grief Inventory-Self Report Plus (TGI-SR+). Comprehensive Psychiatry, 112, 152281. doi: 10.1016/j.comppsych.2021.152281 34700189

[34] Blevins CA, Weathers FW, Davis MT, Witte TK, Domino JL. The Posttraumatic Stress Disorder Checklist for DSM-5 (PCL-5): Development and initial psychometric evaluation: Posttraumatic stress disorder checklist for DSM-5. J Trauma Stress. 2015;28(6):489–98. doi: 10.1002/jts.22059 26606250

[35] Boeschoten M, Bakker A, Jongedijk R, Olff M. Dutch version of the PCL-5. Diemen: ARQ National Psychotrauma Centre. 2014.

[36] American Psychiatric Association. Diagnostic and statistical manual of mental disorders (5th ed.). Arlington, VA: American Psychiatric Publishing. 2013.

[37] Mundt JC, Marks IM, Shear MK, Greist JH. The Work and Social Adjustment Scale: a simple measure of impairment in functioning. Br J Psychiatry. 2002;180:461–4. doi: 10.1192/bjp.180.5.461 11983645

[38] de Graaf LE, Gerhards SAH, Arntz A, Riper H, Metsemakers JFM, Evers SMAA, et al. Clinical effectiveness of online computerised cognitive-behavioural therapy without support for depression in primary care: randomised trial. Br J Psychiatry. 2009;195(1):73–80. doi: 10.1192/bjp.bp.108.054429 19567900

[39] Schwarzer R., & Jerusalem M. (1995). Generalized Self-Efficacy scale. In Weinman J., Wright S., & Johnston M. (Eds.), Measures in health psychology: A user’s portfolio. Causal and control beliefs (pp. 35–37). Windsor, United Kingdom: NFER-NELSON.

[40] Luszczynska A., Scholz U., & Schwarzer R. (2005). The general self-efficacy scale: multicultural validation studies. The Journal of Psychology, 139(5), 439–457. doi: 10.3200/JRLP.139.5.439-457 16285214

[41] Boelen PA. Variables mediating the linkage between loss centrality and postloss psychopathology. J Nerv Ment Dis. 2012;200(9):801–6. doi: 10.1097/NMD.0b013e318266ba02 22922243

[42] Bjureberg J, Ljótsson B, Tull MT, Hedman E, Sahlin H, Lundh L-G, et al. Development and validation of a brief version of the difficulties in Emotion Regulation Scale: The DERS-16. J Psychopathol Behav Assess. 2016;38(2):284–96. doi: 10.1007/s10862-015-9514-x 27239096 PMC4882111

[43] Muthén LK, Muthén BO. Mplus user’s guide (Eighth ed.). Los Angeles, CA: Muthén & Muthén (1998–2019)

[44] Prins A, Bovin MJ, Kimerling R, Kaloupek DG, Marx BP, Pless Kaiser A, et al. The Primary Care PTSD Screen for DSM-5 (PC-PTSD-5). [Measurement instrument]. PTSD: National Center for PTSD [Internet]. Ptsd.va.gov. [cited 2021 Sep 24]. Available from: http://www.ptsd.va.gov/professional/assessment/screens/pc-ptsd.asp

[45] Prigerson HG, Boelen PA, Xu J, Smith KV, Maciejewski PK. Validation of the new DSM-5-TR criteria for prolonged grief disorder and the PG-13-Revised (PG-13-R) scale. World Psychiatry. 2021;20(1):96–106. doi: 10.1002/wps.20823 33432758 PMC7801836

[46] Nylund KL, Asparouhov T, Muthén BO. Deciding on the number of classes in latent class analysis and growth mixture modeling: A Monte Carlo simulation study. Struct Equ Modeling. 2007;14(4):535–69. 10.1080/10705510701575396

[47] Galatzer-Levy IR, Huang SH, Bonanno GA. Trajectories of resilience and dysfunction following potential trauma: A review and statistical evaluation. Clin Psychol Rev. 2018;63:41–55. doi: 10.1016/j.cpr.2018.05.008 29902711

[48] Komischke-Konnerup KB, Zachariae R, Johannsen M, Nielsen LD, O’Connor M. Co-occurrence of prolonged grief symptoms and symptoms of depression, anxiety, and posttraumatic stress in bereaved adults: A systematic review and meta-analysis. Journal of Affective Disorders Reports. 2021;4(100140):100140. 10.1016/j.jadr.2021.100140

[49] Ozer EJ, Best SR, Lipsey TL, Weiss DS. Predictors of posttraumatic stress disorder and symptoms in adults: A meta-analysis. Psychol Bull. 2003;129(1):52–73. doi: 10.1037/0033-2909.129.1.52 12555794

[50] Eisma MC, Schut HAW, Stroebe MS, Boelen PA, van den Bout J, Stroebe W. Adaptive and maladaptive rumination after loss: A three-wave longitudinal study. Br J Clin Psychol. 2015;54(2):163–80. doi: 10.1111/bjc.12067 25229192

[51] Echiverri AM, Jaeger JJ, Chen JA, Moore SA, Zoellner LA. “dwelling in the past”: The role of rumination in the treatment of posttraumatic stress disorder. Cogn Behav Pract. 2011;18(3):338–49. doi: 10.1016/j.cbpra.2010.05.008 22190846 PMC3242379

[52] Eisma MC, Stroebe MS. Emotion regulatory strategies in complicated grief: A systematic review. Behav Ther. 2021;52(1):234–49. doi: 10.1016/j.beth.2020.04.004 33483120

[53] Lanza ST, Rhoades BL. Latent Class Analysis: An alternative perspective on subgroup analysis in prevention and treatment. Prev Sci. 2013 April; 14(2): 157–168. doi: 10.1007/s11121-011-0201-1 21318625 PMC3173585

[54] Eisma MC, Boelen PA, van den Bout J, Stroebe W, Schut HAW, Lancee J, et al. Internet-based exposure and behavioral activation for complicated grief and rumination: A randomized controlled trial. Behav Ther. 2015;46(6):729–48. doi: 10.1016/j.beth.2015.05.007 26520217

[55] Foa EB, Hembree EA, Cahill SP, Rauch SAM, Riggs DS, Feeny NC, et al. Randomized trial of prolonged exposure for posttraumatic stress disorder with and without cognitive restructuring: outcome at academic and community clinics. J Consult Clin Psychol. 2005;73(5):953–64. doi: 10.1037/0022-006X.73.5.953 16287395

