# Supplementary material for: Traumatic stress, depression, and non-bereavement grief following non-fatal traffic accidents: Symptom patterns and correlates
Source: PLoS One. 2022 Feb 28;17(2):e0264497. doi: 10.1371/journal.pone.0264497 (PMC8884715; doi:10.1371/journal.pone.0264497)
Supplement: S1 Table — (DOCX) [file pone.0264497.s002.docx]

Supporting information Table 1

Summary of distinct regression analyses with symptom levels and functional impairment predicting class membership

|  | Reference profile | | | | | | | | | | | | |
| --- | --- | --- | --- | --- | --- | --- | --- | --- | --- | --- | --- | --- | --- |
|  | Class 1: No symptoms | | | | | |  | Class 2: Moderate PTS and grief | | | | | |
| Comparison profile | Est | SE | Exp(B) | 95% CI | | p |  | Est | SE | Exp(B) | 95% CI | | p |
| Class 2: Moderate PTS and grief |  |  |  |  |  |  |  |  |  |  |  |  |  |
| PTS total (PCL-5) | 0.288 | 0.035 | 1.333 | 1.245 | 1.428 | <0.001 |  |  |  |  |  |  |  |
| PTS B cluster items | 0.628 | 0.087 | 1.876 | 1.582 | 2.222 | <0.001 |  |  |  |  |  |  |  |
| PTS C cluster items | 0.739 | 0.098 | 2.096 | 1.730 | 2.528 | <0.001 |  |  |  |  |  |  |  |
| PTS D cluster items | 0.508 | 0.077 | 1.661 | 1.428 | 1.930 | <0.001 |  |  |  |  |  |  |  |
| PTS E cluster items | 0.372 | 0.054 | 1.451 | 1.305 | 1.610 | <0.001 |  |  |  |  |  |  |  |
| Grief (TGI-SR+) | 0.431 | 0.067 | 1.538 | 1.349 | 1.754 | <0.001 |  |  |  |  |  |  |  |
| Depression (HADS-D) | 0.117 | 0.054 | 1.124 | 1.011 | 1.251 | 0.030 |  |  |  |  |  |  |  |
| Functional impairment (WSAS) | 0.185 | 0.036 | 1.203 | 1.121 | 1.290 | <0.001 |  |  |  |  |  |  |  |
| Class 3: Severe symptoms |  |  |  |  |  |  |  |  |  |  |  |  |  |
| PTS total (PCL-5) | 0.454 | 0.053 | 1.574 | 0.573 | 0.705 | <0.001 |  | 0.165 | 0.033 | 1.179 | 1.106 | 1.257 | <0.001 |
| PTS B cluster items | 0.854 | 0.098 | 2.347 | 0.351 | 0.516 | <0.001 |  | 0.226 | 0.046 | 1.253 | 1.144 | 1.371 | <0.001 |
| PTS C cluster items | 0.926 | 0.112 | 2.525 | 0.318 | 0.494 | <0.001 |  | 0.186 | 0.085 | 1.204 | 1.020 | 1.422 | 0.028 |
| PTS D cluster items | 0.988 | 0.152 | 2.688 | 0.277 | 0.501 | <0.001 |  | 0.480 | 0.111 | 1.618 | 1.300 | 2.012 | <0.001 |
| PTS E cluster items | 0.764 | 0.094 | 2.145 | 0.387 | 0.560 | <0.001 |  | 0.392 | 0.079 | 1.481 | 1.267 | 1.730 | <0.001 |
| Grief (TGI-SR+) | 0.571 | 0.081 | 1.769 | 0.482 | 0.663 | <0.001 |  | 0.140 | 0.029 | 1.149 | 1.085 | 1.218 | <0.001 |
| Depression (HADS-D) | 0.836 | 0.115 | 2.309 | 0.346 | 0.543 | <0.001 |  | 0.719 | 0.101 | 2.053 | 1.683 | 2.500 | <0.001 |
| Functional impairment (WSAS) | 0.326 | 0.043 | 1.385 | 0.633 | 0.785 | <0.001 |  | 0.141 | 0.023 | 1.512 | 1.100 | 1.206 | <0.001 |

*Note.* DERS-16 = Difficulties in Emotion Regulation Scale-16. GSES = General Self-Efficacy Scale. HADS = Hospital Anxiety and Depression Scale. PCL-5 = Posttraumatic Stress Disorder Checklist for DSM-5. RQ = Rumination Questionnaire. TGI-SR+ = Traumatic Grief Inventory-Self-Report Plus. WSAS = Work and Social Adjustment Scale.
