# Supplementary material for: Traumatic stress, depression, and non-bereavement grief following non-fatal traffic accidents: Symptom patterns and correlates
Source: PLoS One. 2022 Feb 28;17(2):e0264497. doi: 10.1371/journal.pone.0264497 (PMC8884715; doi:10.1371/journal.pone.0264497)
Supplement: S2 Table — (DOCX) [file pone.0264497.s003.docx]

Supporting information Table 2

Summary of distinct regression analyses with sociodemographic and loss-related variables predicting class membership

|  | Reference profile | | | | | | | | | | | | |
| --- | --- | --- | --- | --- | --- | --- | --- | --- | --- | --- | --- | --- | --- |
|  | Class 1: No symptoms | | | | | |  | Class 2: Moderate PTS and grief | | | | | |
| Comparison profile | Est | SE | Exp(B) | 95% CI | | p |  | Est | SE | Exp(B) | 95% CI | | p |
| Class 2: Moderate PTS and grief |  |  |  |  |  |  |  |  |  |  |  |  |  |
| Gender | -0.106 | 0.303 | 0.899 | 0.496 | 1.628 | 0.726 |  |  |  |  |  |  |  |
| Age | 0.053 | 0.012 | 1.054 | 1.029 | 1.078 | <0.001 |  |  |  |  |  |  |  |
| Education (0=low, 1=high) | 1.064 | 0.297 | 2.898 | 1.621 | 5.181 | <0.001 |  |  |  |  |  |  |  |
| Months since accident | 0.003 | 0.001 | 1.003 | 1.000 | 1.059 | 0.038 |  |  |  |  |  |  |  |
| Transportation vehicle (0=other,  1=car/motorcycle | 0.251 | 0.289 | 1.285 | 0.729 | 2.267 | 0.385 |  |  |  |  |  |  |  |
| Were you driver? (0=no, 1=yes) | 0.490 | 0.325 | 1.631 | 0.823 | 3.086 | 0.132 |  |  |  |  |  |  |  |
| Perceived threat to life | 0.272 | 0.066 | 1.312 | 1.155 | 1.492 | <0.001 |  |  |  |  |  |  |  |
| Physical injury (0=no, 1=yes) | 1.660 | 0.392 | 5.263 | 2.439 | 11.363 | <0.001 |  |  |  |  |  |  |  |
| Class 3: Severe symptoms |  |  |  |  |  |  |  |  |  |  |  |  |  |
| Gender | -0.192 | 0.317 | 0.825 | 0.443 | 1.536 | 0.545 |  | -0.086 | 0.376 | 0.917 | 0.439 | 1.915 | 0.819 |
| Age | 0.071 | 0.011 | 1.074 | 1.050 | 1.097 | <0.001 |  | 0.018 | 0.009 | 1.018 | 1.033 | 1.035 | 0.032 |
| Education (0=low, 1=high) | 0.573 | 0.315 | 1.773 | 0.956 | 3.289 | 0.069 |  | -0.491 | 0.363 | 0.612 | 0.300 | 1.246 | 0.176 |
| Months since accident | 0.005 | 0.001 | 1.005 | 1.002 | 1.072 | <0.001 |  | 0.002 | 0.002 | 1.002 | 0.998 | 1.005 | 0.315 |
| Transportation vehicle (0=other,  1=car/motorcycle | -0.150 | 0.303 | 0.861 | 0.475 | 1.557 | 0.620 |  | -0.402 | 0.362 | 0.669 | 0.329 | 1.360 | 0.267 |
| Were you driver? (0=no, 1=yes) | 1.368 | 0.440 | 3.921 | 1.658 | 9.259 | 0.002 |  | 0.878 | 0.502 | 2.410 | 0.899 | 6.451 | 0.080 |
| Perceived threat to life | 0.340 | 0.077 | 1.404 | 1.208 | 1.634 | <0.001 |  | 0.067 | 0.087 | 1.069 | 0.902 | 1.267 | 0.437 |
| Physical injury (0=no, 1=yes) | 2.265 | 0.386 | 9.615 | 4.524 | 20.408 | <0.001 |  | 0.606 | 0.373 | 1.831 | 0.881 | 3.802 | 0.104 |
