# Supplementary material for: Traumatic stress, depression, and non-bereavement grief following non-fatal traffic accidents: Symptom patterns and correlates
Source: PLoS One. 2022 Feb 28;17(2):e0264497. doi: 10.1371/journal.pone.0264497 (PMC8884715; doi:10.1371/journal.pone.0264497)
Supplement: S3 Table — (DOCX) [file pone.0264497.s004.docx]

Supporting information Table 3

Summary of distinct regression analyses with self-efficacy, difficulties in emotion regulation, and rumination predicting class membership

|  | Reference profile | | | | | | | | | | | | |
| --- | --- | --- | --- | --- | --- | --- | --- | --- | --- | --- | --- | --- | --- |
|  | Class 1: No symptoms | | | | | |  | Class 2: Moderate PTS and grief | | | | | |
| Comparison profile | Est | SE | Exp(B) | 95% CI | | p |  | Est | SE | Exp(B) | 95% CI | | p |
| Class 2: Moderate PTS and grief |  |  |  |  |  |  |  |  |  |  |  |  |  |
| Self-efficacy | -0.397 | 0.296 | 0.672 | 0.376 | 1.200 | 0.180 |  |  |  |  |  |  |  |
| Difficulties emotion regulation | -0.006 | 0.013 | 0.994 | 0.969 | 1.019 | 0.641 |  |  |  |  |  |  |  |
| Trauma rumination | 0.676 | 0.127 | 1.964 | 1.533 | 2.525 | <0.001 |  |  |  |  |  |  |  |
| Class 3: High symptoms |  |  |  |  |  |  |  |  |  |  |  |  |  |
| Self-efficacy | -1.565 | 0.378 | 0.209 | 0.099 | 0.438 | <0.001 |  | -1.168 | 0.397 | <0.001 | 0.143 | 0.677 | 0.003 |
| Difficulties emotion regulation | 0.053 | 0.011 | 1.055 | 1.031 | 1.077 | <0.001 |  | 0.059 | 0.015 | 1.060 | 1.030 | 1.092 | <0.001 |
| Trauma rumination | 0.948 | 0.142 | 2.958 | 1.953 | 3.413 | <0.001 |  | 0.272 | 0.065 | 1.312 | 1.154 | 1.490 | <0.001 |
