# Supplementary material for: Traumatic stress, depression, and non-bereavement grief following non-fatal traffic accidents: Symptom patterns and correlates
Source: PLoS One. 2022 Feb 28;17(2):e0264497. doi: 10.1371/journal.pone.0264497 (PMC8884715; doi:10.1371/journal.pone.0264497)
Supplement: S4 Table — (DOCX) [file pone.0264497.s005.docx]

Supporting information Table 4

Summary of regression analyses with sociodemographic and loss-related variables, self-efficacy, difficulties in emotion regulation, and trauma rumination (entered simultaneously) predicting class membership

|  | Reference profile | | | | | | | | | | | | |
| --- | --- | --- | --- | --- | --- | --- | --- | --- | --- | --- | --- | --- | --- |
|  | Class 1: No symptoms | | | | | |  | Class 2: Moderate PTS and grief | | | | | |
| Comparison profile | Est | SE | Exp(B) | 95% CI | | p |  | Est | SE | Exp(B) | 95% CI | | p |
| Class 2: Moderate PTS and grief |  |  |  |  |  |  |  |  |  |  |  |  |  |
| Age | 0.020 | 0.018 | 1.020 | 0.984 | 1.056 | 0.272 |  |  |  |  |  |  |  |
| Education (0=low, 1=high) | 0.408 | 0.493 | 1.524 | 0.572 | 3.952 | 0.407 |  |  |  |  |  |  |  |
| Months since accident | 0.001 | 0.003 | 1.001 | 0.996 | 1.007 | 0.695 |  |  |  |  |  |  |  |
| Were you driver? (0=no,1=yes) | 0.358 | 0.480 | 1.430 | 0.558 | 3.663 | 0.456 |  |  |  |  |  |  |  |
| Perceived threat to life | 0.195 | 0.102 | 1.215 | 0.995 | 1.485 | 0.056 |  |  |  |  |  |  |  |
| Physical injury (0=no, 1=yes) | 0.664 | 0.616 | 1.941 | 0.581 | 6.493 | 0.281 |  |  |  |  |  |  |  |
| Self-efficacy | -1.302 | 0.572 | 0.272 | 0.089 | 0.502 | 0.023 |  |  |  |  |  |  |  |
| Difficulties emotion regulation | 0.002 | 0.020 | 1.002 | 0.963 | 1.043 | 0.920 |  |  |  |  |  |  |  |
| Trauma rumination | 0.579 | 0.147 | 1.785 | 1.338 | 2.380 | <0.001 |  |  |  |  |  |  |  |
| Class 3: Severe symptoms |  |  |  |  |  |  |  |  |  |  |  |  |  |
| Age | 0.064 | 0.026 | 1.066 | 1.013 | 1.121 | 0.014 |  | 0.044 | 0.020 | 1.044 | 1.005 | 1.085 | 0.025 |
| Education (0=low, 1=high) | -0.549 | 0.664 | 0.577 | 0.157 | 2.123 | 0.408 |  | -0.957 | 0.552 | 0.383 | 0.130 | 1.131 | 0.083 |
| Months since accident | 0.002 | 0.003 | 1.002 | 0.996 | 1.008 | 0.545 |  | 0.001 | 0.002 | 1.001 | 0.997 | 1.004 | 0.706 |
| Were you driver? (0=no,1=yes) | 1.405 | 0.654 | 4.081 | 1.131 | 14.705 | 0.032 |  | 1.047 | 0.646 | 2.849 | 0.803 | 10.101 | 0.105 |
| Perceived threat to life | 0.277 | 0.148 | 1.319 | 0.988 | 1.763 | 0.061 |  | 0.082 | 0.131 | 1.085 | 0.839 | 1.402 | 0.531 |
| Physical injury (0=no, 1=yes) | 0.809 | 0.777 | 2.247 | 0.489 | 10.309 | 0.298 |  | 0.145 | 0.686 | 1.156 | 0.301 | 4.444 | 0.832 |
| Self-efficacy | -2.124 | 0.667 | 0.119 | 0.032 | 0.442 | <0.001 |  | -0.822 | 0.564 | 0.439 | 0.145 | 1.328 | 0.145 |
| Difficulties emotion regulation | 0.096 | 0.027 | 1.100 | 1.043 | 1.160 | <0.001 |  | 0.094 | 0.029 | 1.097 | 1.038 | 1.122 | <0.001 |
| Trauma rumination | 0.922 | 0.156 | 2.512 | 1.851 | 3.412 | <0.001 |  | 0.343 | 0.098 | 1.408 | 1.161 | 1.706 | <0.001 |
