# Supplementary figures and images for: Traumatic stress, depression, and non-bereavement grief following non-fatal traffic accidents: Symptom patterns and correlates
Source: PLoS One. 2022 Feb 28;17(2):e0264497. doi: 10.1371/journal.pone.0264497 (PMC8884715; doi:10.1371/journal.pone.0264497)

Supporting information Figure 1

Flowchart of participants


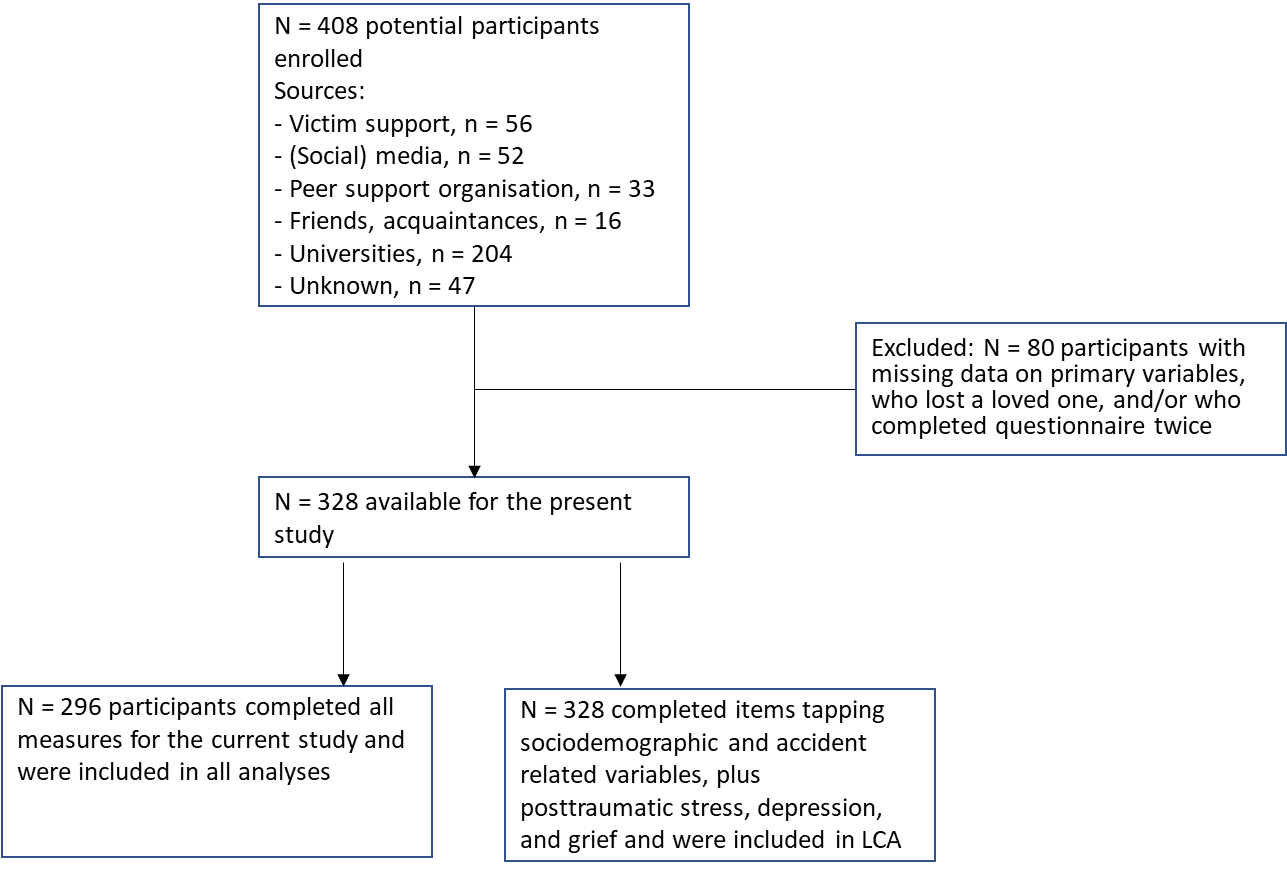

Supplement: S1 Fig — (DOCX) [file pone.0264497.s006.docx]
